# Views of Nigerian civil servants about compulsory COVID-19 vaccination: A qualitative study

**DOI:** 10.4102/phcfm.v16i1.4208

**Published:** 2024-02-23

**Authors:** Nyemike S. Awunor, Luret A. Lar, Alphonsus R. Isara

**Affiliations:** 1 Department of Community Medicine, Faculty of Clinical Sciences, Delta State University, Abraka, Nigeria; 2 Department of Community Medicine, Delta State University Teaching Hospital, Oghara, Nigeria; 3 Department of Community Medicine, Faculty of Clinical Sciences, University of Jos, Jos, Nigeria; 4 Department of Community Medicine, Jos University Teaching Hospital, Jos, Nigeria; 5 Department of Community Health, Faculty of Medical Sciences, University of Benin, Benin, Nigeria

**Keywords:** COVID-19 vaccination, compulsion, civil servants, qualitative study, Nigeria

## Abstract

**Background:**

COVID-19 caused unforeseen global burden, although vaccine strategy rapidly stalled transmission and protected those at risk. Many governments made vaccination mandatory for public space access.

**Aim:**

This study aimed to elucidate perception of Nigerian civil servants towards mandatory COVID-19 vaccination and elicited their recommendations.

**Setting:**

This study was conducted in twelve purposively selected states in the six geopolitical zones and the Federal Capital Territory (FCT), Nigeria. Relevant ministries, departments and agencies were selected within the study sites.

**Methods:**

It was a qualitative study that interviewed consenting civil servants. Ethical approval was obtained from the National Health Research Ethics Committee. Interviews were conducted in person, following a pre-test. Data was analysed using NVivo software version 12.

**Results:**

Most participants were willing to take the vaccine if their safety was assured. However, enforcement to do so was a hindrance. Most participants commended the government for the effort to curb COVID-19 transmission and create awareness but were displeased with planning and handling of misconceptions. They recommended a more committed approach to vaccine production and funding by the government.

**Conclusion:**

Participants were willing to take the COVID-19 vaccines because the gains of protection outweighed the risks. They suggested a less involuntary approach through reinforcing awareness creation and avoiding threats.

**Contribution:**

There is limited qualitative research on perception of Nigerian civil servants regarding mandatory COVID-19 vaccination. Being the main driving force of Nigeria’s public service, their views are invaluable. Findings could contribute to future policies in times of emergency.

## Introduction

The coronavirus disease 2019 (COVID-19) pandemic, caused by the new coronavirus SARS-CoV-2, has had an extraordinary impact on global public health, and appears to be one of the most current substantial international challenges.^1^ Since its original documentation in late 2019, the virus has rapidly spread across borders, leading to extensive illnesses, hospital admissions, and mortalities.^2^ Modelling conducted in Nigeria showed that travelling and contacts increased infection rates by 85% and 88%, respectively.^3^ The magnitude of the pandemic demanded an inclusive and multidimensional approach to mitigate its devastating effects.

As countries and communities contended with the burden of the pandemic, scientists and healthcare professionals persistently worked to develop effective approaches to control the transmission of COVID-19 and to protect susceptible people.^4^

Among such vital interventions, the development and distribution of vaccines against the virus are a fundamental landmark in the global response to the pandemic.^5^ Nigeria was the third West African country to obtain COVAX vaccines, succeeding Ghana and Ivory Coast in March 2021.^6^ Approximately 8 million Nigerians were vaccinated during the first and second phases of the campaign.^7^

However, despite outstanding advancement in vaccine development and distribution, challenges remain in reaching the anticipated levels of population immunity.^8,9^ Vaccine hesitancy, misinformation, and appearance of new viral variants created hindrances to successful control of the pandemic.^8,10,11^ Consequently, 80% of Nigerians were unwilling to participate in COVID-19 vaccine trial due to some of these challenges.^12^ Therefore, some governments and organisations implemented mandatory vaccination policies that entailed eligible individuals to take the COVID-19 vaccine.^13^ Nigeria was not left behind, as the Federal Government announced on 01 December 2021, that all civil servants must produce a proof of COVID-19 vaccination or negative polymerase chain reaction (PCR) test to be allowed access to their offices.^12^

Compulsory vaccination guidelines have created debates around human rights, ethical issues and poise between public health and individual decisions.^14,15,16,17^ Consequently, appreciating the justification, effect, and consequences of obligatory COVID-19 vaccination is critical for policymakers, healthcare providers, and the public.^18^ Therefore, this study aimed to explore the rationale around mandatory COVID-19 vaccination among Nigerian civil servants, explore its effect on public health outcomes, and critically assess the ethical deliberations related with such policies.

## Methods

### Study design

This was a qualitative study that employed in-depth interviews (IDIs). This epistemological approach was taken to understand realities of participants’ experiences, through the social constructionist lens, especially that such experiences are subjective.^19^

### Setting

**FIGURE 1:**
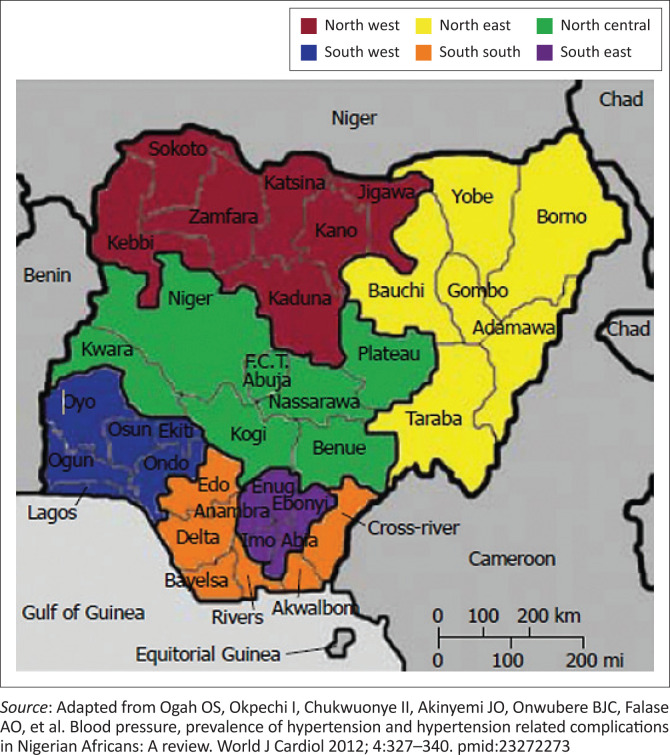
Map of Nigeria showing the geopolitical zones and country borders.

The national study was conducted across the six geopolitical zones (GPZs) of the country and the Federal Capital Territory (FCT), Abuja (Figure 1).^20^ The research team purposively selected two states from each of the GPZs, totalling 12 states across the federation, and the FCT (Table 1). The study duration was 6 months, from July to December 2022.

**TABLE 1:** States included in the study and the Federal Capital Territory.

Geopolitical zone	State
North central	Benue
	Plateau
North east	Borno
	Gombe
North west	Kaduna
	Kano
South east	Enugu
	Imo
South-south	Cross River
	Delta
South west	Ondo
	Oyo
FCT Abuja	-

FCT, Federal Capital Territory.

Nigeria is commonly referred to as the ‘giant of Africa’, with a current population of over 200m.^21,22,23^ and has a projected population of 264m in 2030.^24^ The country is ethnically and culturally diverse and the economy is largely driven by the oil sector and a growing agricultural division.^25^ While Nigeria has experienced outbreaks of several infectious diseases, such as Lassa fever and Ebola virus disease in 2014, no pandemic has originated solely in Nigeria.^26,27^ Sadly, the weak healthcare system insufficiently supports such pandemics.^28,29^

### Study population and sampling strategy

Permanent Secretaries (PS) or their representative in the Ministries, Departments and Agencies (MDAs) were purposively invited to participate in the IDIs. At least two participants were separately interviewed in each State and in the FCT. Purposive sampling was also used to select at least one Chairman or their representative of a Labour Union in each State, including the FCT.

### Data collection

Qualitative data were collected in English using a semi-structured interview guide with 10 questions and probes. The questions included information sources regarding COVID-19 vaccination, availability of the vaccines, perceptions of civil servants about the level of vaccination and mandatory vaccination, misinformation and myths regarding the vaccines, factors affecting vaccine uptake, handling of the vaccinations by the government, and suggestions for improvement. An example of a specific question and probe used was: (1) What are the factors influencing the general level of vaccination observed among civil servants in the state? Probe: What is the role of availability of the COVID-19 vaccines? In what ways do conspiracy theories and misinformation affect the vaccination drive?

The interview guide was developed from findings of related published articles.^14,30,31,32^ The guides were pre-tested among civil servants in MDAs in Benin City, Edo State to correct for any ambiguities, such as making unclear questions more explicit, and to determine the average time required to conduct the interview, which ranged between 30 and 50 min. The findings of these pretested interviews were not included in the final study. Audio-recorded interviews were conducted in person by trained researchers who were predominantly Resident Doctors with knowledge and experience in conducting qualitative research. The interviews lasted between 20 min and 45 min. A total of 24 interviews were conducted (Table 3) at the point where nothing new was emerging from the data, thereby indicating data saturation.^33^

### Data analysis

The interviews were recorded and where feasible, recorded data were transcribed verbatim and notes were taken for quality control, to document emerging findings and to capture reflections.^34^ Grounded theory approach^35^ was employed as data collection and analysis were simultaneously conducted, thereby producing inductive themes, rather than using existing frameworks to deductively analyse the data. The iterative process of comparative analysis was employed using initial open coding, where descriptive codes were assigned. The authors then applied axial coding for a more integrated theory and then they used the selective coding to unify these emergent themes around the perception of civil servants about the mandatory COVID-19 vaccination.^35,36^

A coding framework was developed by the research team after familiarisation with the data. Attributes used for further analysis included cadre, gender, GPZ, and vaccination status. NVivo software (QSR International Pty Ltd.) version 12 plus, 2018 was used to organise the data, which was summarised into overarching themes using thematic framework approach.^37^ Researchers declared their positionality; member checking was done at the end of each interview to ensure that what the participants mentioned was correctly documented and deviant responses were included to maintain the trustworthiness and credibility of the data.^38,39,40^

### Ethical considerations

Ethical approval was obtained from the Federal Ministry of Health, National Health Research Ethics Committee (No. NHREC/01/01/2007–22/06/2022.). Permissions were obtained from State MDAs. Both verbal and written informed consent was obtained from the participants prior to data collection. The written consents were collected in duplicate, with the participants and the research team each retaining copies, in case of any legal issues.^41^ Privacy of participants was ensured by conducting the interviews in their preferred locations, so that they were free to express themselves. They were informed to decline from answering questions that they were uncomfortable with. To ensure anonymity and data confidentiality, quotes were inconspicuously described using code identifiers (Table 3).

## Results

Seven main themes emerged, as discussed below: (1) Knowledge of COVID-19 Among Civil Servants, (2) Willingness to be Vaccinated, (3) Handling of COVID-19 by Government, (4) Perception and Attitude of Civil Servants Towards the Vaccine Mandate, (5) Facilitators of Mandatory COVID-19 Vaccination, (6) Barriers of Mandatory COVID-19 Vaccination and (7) Recommendations for Uptake of COVID-19 Vaccination. The identifiers shown in Table 3 were used to qualify the quotes, making the characteristics of the participant more explicit. Most civil servants were knowledgeable about the pandemic and the vaccine, with many of them taking at least one dose of the vaccine. They had mixed feelings about how the government handled the pandemic.

**TABLE 2:** Sociodemographic details of participants.

Serial number	Geopolitical zone	Variable	Ministry and/or agency
1	South south	Assistant Directors and Union Leader	State Ministry of Education, Nigeria Union of Teachers, and State Ministry of Environment
2	South east	Administration staff and Deputy Director	Independent National Electoral Commission (INEC), Local Government Area (LGA) Civil Service Commission, Ministry of Justice, News Agency of Nigeria
3	South west	Permanent Secretaries, Secretaries, Chairmen	Ministry of Education, Nigeria Labour Congress (NLC), LGA Nigeria Union of Local Government Employees (NULGE)
4	North east	Chairmen and Directors	NLC, Ministry of Education
5	North central	Union Leaders, and Deputy Directors	Ministry of Health
6	North west	Directors Chief Education Officer	Ministry of Health, Ministry of Higher Education
7	FCT	Directors Union Leaders, Human Resources Managers	Federal Capital Territory Administration (FCTA) Nigeria Union of Teachers
8	Sex	Majority of the participants were male	-
9	Age (years)	This ranged between 42 and 59	-
10	Years of experience	This ranged between 5 and 34 years	-
11	Number of IDIs	24	-

FCT, Federal Capital Territory; IDI, in-depth interviews.

**TABLE 3:** Code identifiers.

Serial number	Attribute	Code	Description
1	Geo-political zone	NC	Northcentral
		NE	Northeast
		NW	Northwest
		SE	Southeast
		SS	South south
		SW	Southwest
2	Gender	F	Female
		M	Male
3	Cadre	ADC	Administrative class
		EC	Executive class
		PC	Professional class
		CC	Clerical class
		AC	Auxiliary class
4	Vaccination status	FV	Fully Vaccinated
		NS	Not stated
		NV	Not vaccinated
		PV	Partially vaccinated

### Knowledge of COVID-19 among civil servants

Majority of civil servants were knowledgeable and possessed some appreciable information about COVID-19. This awareness was provided by the government at the peak of the pandemic and lockdown. Furthermore, there were various protocols, such as social distancing, wearing facemasks and handwashing campaigns. This was well publicised in both electronic and print media, as well as on social media sites, such as Facebook and Twitter. However, a few participants did not have vast and specific knowledge of the pandemic, apart from being aware that there was a lockdown and some restrictions due to the outbreak of the pandemic:

‘Effective; the people concerned, governmental agencies and non-governmental bodies have done justice in information dissemination across the State. One or two circulars were also released from the office of the Head of Service on the directive of Mr. Governor as a matter of policy mandating every civil servant to be vaccinated. That means all MDAs must have been informed and domesticated same in their various ministries and among their members of staff so I want to say that in terms of figure now, let’s put it at 85 to 90% of civil servants in the State must or should have known or heard about COVID-19 and what is expected of them to do.’ (EC, M, SW, FV)

### Willingness to be vaccinated

Most civil servants across all cadres were willing to be vaccinated because they were enlightened and educated people who placed value on their health and that of others:

‘I am fully vaccinated, I have taken my first, second and booster doses.’ (AC, M, SS, FV)

‘With adequate information and the right knowledge being pushed into the minds of our people, I want to say they are willing. For instance, we held a program by the implementation team on COVID-19 which was launched in Ondo State on the 16th and 17th of last month [*July*]. This team comprised of NACA, NLC, NCDC, PHCDA and MOH. We held a 2-day program at the Royal Bird Hotel [*Akure*] where we inaugurated the State implementation team and could you believe that in that program, after the inauguration those who have not been vaccinated willingly came forward for vaccination. Willingly, those who have taken the first came out to take the second and those who have taken the second also took the booster jab. So, with the right knowledge, right information, people will willingly come out.’ (EC, M, SW, FV)

In addition to being enlightened about the pandemic, several civil servants complied with mandatory vaccination once they were assured of the safety of the vaccine:

‘Well as far as I know they brought this thing down here to us in the offices. Although I don’t know about the public places because I have not been there, almost 99% of us in the office here are vaccinated. I have had conversations with some of my friends in different parastatals and I have been told they got vaccinated with even their second jab.’ (EC, M, SE, FV)

Few unwilling civil servants remained unvaccinated because of inadequate information and education about the pandemic as well as the vaccine. However, the quotes below support this finding:

‘Yes, it is low; I will use my office as an instance, I had to beg people to take the vaccine. Out of 20 in my office, only six agreed to get vaccinated to the extent that I made efforts to get the vaccination team to come to my office, but they refused. This happened in other places too because I also monitored. I know that people have some religious beliefs and all those superstitious stories. That is what is discouraging them from taking the vaccine.’ (IDI, AC, F, SE, FV)

‘… the level is not optimal, in the sense that because of the information that they got, that a lot of things came; a lot of benefits came from COVID-19 and few section of civil servants who were handling it directly benefitted from it and the others did not benefit from it. So, because of that, there was apathy. Yeah, very low.’ (IDI, AC, M, SS, FV)

### Handling of COVID-19 by government

Most civil servants mentioned that the government had done well on handling the COVID-19 pandemic, given its sudden appearance on the scene and the limited resources that the government had for an emergency of that magnitude. Successes included setting up a task force to respond to the pandemic and provision of policy guidelines, establishing a protocol to be followed for risk mitigation and communication, provision of allowances for workers at the frontline of containing the pandemic, and adequate provision of testing centres:

‘… we have steering committees at this level, and later which transform to the presidential task force to the steering committee, you know they were really very effective when looking at how the country will cope up and also at the state level.’ (ADC, M, NW, FV)

However, despite commendations from some civil servants, many of them did not score the government high. The reasons given were the haphazard handling of the situation, being inadequately proactive, and low response or widespread corruption in their efforts to mitigate the suffering of civil servants caused by the pandemic. Additionally, information management to counter widespread fear and misconceptions has been poorly handled by the government:

‘Well, from pragmatic aspect of it, we look at the COVID-19 coordination and they have wonderful policies and guidelines.’ (ADC, M, NW, FV)

### Perception and attitude of civil servants towards the vaccine mandate

Although civil servants agreed that the government should protect citizens, they mentioned that the vaccination should be voluntary and not coerced. Making the vaccine mandatory was seen as a violation of the fundamental rights of citizens to choose their own health-seeking behaviour. One unintended consequence of the mandatory vaccination was that those who did not wish to be vaccinated but felt threatened by the position of government on no salaries without vaccination tried to bribe health officials to obtain the vaccine certificate without being vaccinated. This puts innocent citizens at risk:

‘I will say they shouldn’t have made it mandatory because people have different reasons why they will not want to take it. Some may say their faith [*religious beliefs*] does not accept vaccination. Some may not react well to it, some may not even want it at all, and others may want it anyway, but it is not good to make it mandatory seeing that everyone has their own rights. And when someone takes the vaccine and dies it means you have taken away the person’s right to live. So, making it mandatory is not a very good option, they should have made it optional.’ (EC, F, SE, NV)

Many civil servants said that honest, intensive, and continuous sensitisation and education about the COVID-19 pandemic was a better alternative to being mandated to take the vaccine. It is better for people to be convinced than to be coerced. However, some sections of the civil servants thought that the COVID-19 vaccine mandate was proper as it was the responsibility of the government to protect the larger population from anything that may endanger their health and their lives.

### Facilitators of mandatory COVID-19 vaccination

Enablers of willingness to be vaccinated included availability of vaccines, multiple vaccination sites, increased government engagement, education of the citizenry, and political will to follow through with the COVID-19 vaccination mandate. Some civil servants feared contracting the disease in the absence of any known alternative or immunity, thereby facilitating the uptake of the vaccination mandate:

‘… some people don’t believe that COVID-19 exists. The level fact that this disease has not killed so many people in the State, people said it is for the rich people. Well, it affects it in so many ways. One it could be traditional; it could be religious. So, it also depends on the perception of the people, the awareness that was given by who, who gave the awareness and how does the message go. Those things also matter. Yes, some said their productive, their productivity, as a man you won’t be able to produce. It also affects, they said a woman cannot produce also.’ (EC, M, NC, FV)

Majority of civil servants agreed that the potential benefit of mandatory vaccination was curtailing the spread of COVID-19, thereby leading to the safety or health of the people. Additionally, accepting to take the vaccine made it easier to embark on international travel without fear of embarrassment by relevant authorities at various airports or entry points. Moreover, it safeguarded any future consequences from organisations or governments that may attach certain benefits to the evidence of vaccination:

‘I think the major advantage is that it will curtail the effect of the spread of diseases.’ (AC, M, SW, FV)

### Barriers of mandatory COVID-19 vaccination

Participants mentioned improper planning and system-related failures by the government and inadequate handling of widespread misconceptions and misinformation in the mass media as barriers to the vaccination mandate. Furthermore, there was fear of the unknown, including side effects from the vaccine and fear of death, because of several reports about people who ‘died’ after taking the vaccine. Other barriers included religious beliefs, vaccine hesitancy, unavailability of vaccines, a lack of trust in the government and commercial interests of competing pharmaceutical companies:

‘Yes … Misinformation, I say creates fear, that is what mitigates against, accessibility or accessing the vaccine because people saw it, the social media, they were hearing the jingles that if you take it, you will die, that is what causes it.’ (ADC, M, FCT, FV)

### Recommendations for uptake of COVID-19 vaccination

Participants suggested that the government should be proactive and responsible for initiating a robust approach to properly implement, monitor, and supervise the COVID-19 vaccination protocol to ensure compliance. Local production of vaccines through improved funding from research institutes and local scientists should be pursued to compensate for shortages and increase the confidence and trust of the population in the vaccine. Similarly, there should be increased funding for health, including attractive allowances for health workers and implementers on the frontline:

‘Generally, our government in Nigeria should equip our research institutes to enable them to see how we can locally generate and manufacture some of these vaccines, because we don’t know how these things play out in the global village. I guess I am not against vaccination; I am rather saying that our scientists should know what is inside the vaccine and if you don’t know what is inside the vaccine, we shouldn’t be too quick to accept because it could be a Greek gift in disguise, that is all I have to say, thank you.’ (EC, M, SE, NV)

Giving a legal backing for vaccination is also required for increased compliance. A comprehensive and encompassing sensitisation and awareness campaign using all government and community structures is also necessary. Available vaccines and qualified personnel to administer vaccines must be prioritised. State actors should ensure that there is more synergy among them to forge a common front towards the attainment of mandatory vaccination. Furthermore, security should be enhanced to improve the accessibility to vulnerable and difficult-to-reach areas.

## Discussion

### Summary of findings

Referring back to the grounded theory approach^42^ that was used to analyse the findings, the core emerging category was that civil servants generally disagreed with mandatory COVID-19 vaccinations. This theme was used to make linkages with the six other emerging themes that formed the basis of the discussion of the findings. The findings show a good knowledge of the virus, including signs and symptoms, transmission, prevention and control. Although most participants were willing to take the vaccine, a few were unwilling to. There were mixed views about the government’s efforts in tackling the pandemic. However, even as participants felt that the government has the mandate to protect the lives of citizens, coercion was the wrong approach to ensuring that civil servants get vaccinated. Participants suggested that civil servants should be encouraged by the government to do so through sustained awareness creation. They recommended better funding, improving incentives for health workers, accessibility of the vaccines to remote areas and in-country vaccine production. In this discussion, there was a paucity of qualitative research regarding thematic areas that emerged in this study’s findings. Therefore, most comparisons are across non-qualitative studies.

### Comparisons of findings

Good knowledge of COVID-19 in this study was like several other studies.^43,44,45,46^ For example, in a study conducted among the Indian population, 58.6%^47^ of them had good knowledge regarding the cause and/or symptoms, transmission, prevention, treatment and/or care-seeking, and risk of the virus, which were similar to the findings in the current study. This could be because this global pandemic caused governments to create awareness using several channels, especially based on considerations of the fatality of the disease. The media, health facilities, schools, religious places of worship, workplaces and other public spaces were populated with information, and in most instances, in the languages and formats that people could understand. Even when unwilling to hear the information, it was everywhere, and people had no choice but to be confronted with the messaging.

A qualitative study conducted among students in the United States of America (USA) also reported misinformation about the virus and the vaccine, which increased stress among the participants. However, similar to the current study, the effort by the university in testing, establishing on-campus quarantine and isolation spaces were stated to prominently decrease stress and raise perceived safety.^48^ This was like the current study where the effort of the government at tackling the pandemic proactively was commended by study participants. However, in a global survey to assess and score governments, the mean national scores were from 35.76 (Ecuador) to 80.48 (China) out of a maximum of 100 points. Nigeria scored 46.32,^49^ which resonates with the qualitative findings of this study.

Most of the studies conducted in Nigeria around knowledge of the virus were among healthcare workers, and few were qualitative studies.^50,51,52^ One of the studies documented low knowledge scores among healthcare workers with the community health workers scoring less than the facility-based workers.^50^ This finding may not be surprising, because the capacity at the community levels is usually lower than at higher levels of service delivery in Nigeria. However, the findings in the current study were contrary to these findings. Another study conducted among healthcare workers on their knowledge about prevention of the virus was also similar to this study as 82.4% of them had good knowledge.^52^

In a cross-sectional study conducted among Nigerians from the north, east and west GPZ and from varied occupations, over 60% of them were willing to take the COVID-19 vaccines if recommended by health workers.^53^ This finding was in line with the current study finding and can be extrapolated to mean that participants felt safe to take the vaccine if recommended by personnel that they felt could guarantee their safety. Indeed, the effect of COVID-19 vaccination programmes on disease transmission, and its burden on mortality greatly depends on people’s willingness to accept the vaccine. Therefore, like a cross-sectional study conducted in Ghana, which lies in the same West African region as Nigeria, only 21% of the respondents were unlikely to take the vaccine.^54^ This corroborated with the current study.

Employer-mandated vaccinations in most states in the USA are documented to be rejected.^55^ Likewise, mandatory vaccinations could be authorised in numerous sectors based on legal and ethical considerations.^56^ These two statements agree with the findings of this study. Furthermore, Italy was the first country that imposed mandatory vaccinations for its healthcare workers and although 35% of them did not oblige, their reasons revolved around ethical and legal contemplations.^57^ However, the benefits and risks of taking a mandatory approach to vaccination, especially the COVID-19 vaccination, are situational and should be weighed against human rights infringements and risk assessments. A study conducted in Cyprus showed that only 27.8% of participants favoured mandatory vaccinations and this increased with age.^58^ Therefore, policy makers should take careful consideration in making policies in these heterogenous contexts.

## Recommendations and implications for future research

The key recommendation from this study is the need for governments to carefully reconsider mandatory vaccinations, even in the face of emergencies like the COVID-19 pandemic. Many uncertainties surrounded the situation at that time. Recognising the gains of getting citizens vaccinated and protecting those at risk, who may not be eligible for vaccinations is pertinent. However, the approach should be voluntary and more encouraging, rather than using enforcement and threats. This is especially true for a socioculturally diverse country like Nigeria where misinformation and misconceptions around diseases spread rapidly due to ignorance and people’s levels of understanding. Furthermore, structures like the community and religious leaders could have been more engaged to foster awareness, demonstrate by example and win the buy-in of the communities.

Further research could expand to other public and private organisations in the country, so that a comparison across these key sectors could be made. This could be triangulated with focus group discussions from relevant community members about strategies that the government can take to mitigate future occurrences by involving community voices in policy decisions, even in the face of emergencies.

### Strengths and limitations of the study

Perspectives of stakeholders across all GPZs in the country and the FCT were considered. This strengthened the experiences that were shared in the findings. Although this study was qualitative, interviewing stakeholders across all cadres of the civil service and taking into consideration gender dynamics provided maximum variation in the viewpoints.

However, social desirability bias, whereby participants gave responses that they felt will suit the expectations of the researchers could have occurred.^59^ However, other deviant views^38,39^ where participants freely criticised the mandatory COVID-19 vaccination mandate, were captured in the quotes, which most likely minimised this limitation. Missing data were recorded in the analysis and attempts to retrieve such data were not possible. Hence, a table of quotes which attempted to capture such perceptions in order not to omit these findings from the study is available.

## Conclusion

This study showed that most Nigerian civil servants are willing to take the COVID-19 vaccine. However, they are willing to do so by their informed and voluntary choice. Enforcing the vaccines on them could infringe on their fundamental human rights. Therefore, policy makers should explore ethical and legal implications of involuntary guidelines that are implemented on the workforce, especially during emergencies. Considerations should be made to involve citizens appropriately in such decisions.
